# Effects of diaphragmatic paresis following lung transplantation

**DOI:** 10.1186/2197-425X-3-S1-A894

**Published:** 2015-10-01

**Authors:** S Ramirez-Estrada, J Riera, J Baldirà, C Mazo, C Maldonado, C Berastegui, I Bello, T Pont, J Rello, Vall d'Hebron Lung Transplant Study Group Investigators

**Affiliations:** Critical Care, Vall d'Hebron University Hospital-VHIR, Barcelona, Spain; Medicina, Universitat Autònoma de Barcelona, Barcelona, Spain; Instituto de Salud Carlos III, CIBERES, Barcelona, Spain; Pneumology, Vall d'Hebron University Hospital-VHIR, Barcelona, Spain; Thoracic Surgery, Vall d'Hebron University Hospital-VHIR, Barcelona, Spain; Transplant Coordination Department, Vall d'Hebron University Hospital-VHIR, Barcelona, Spain

## Introduction

Early survival after lung transplantation (LT) has improved in the last years. Recent advances in bedside lung ultrasound in the critically ill patient facilitate the diagnosis of diaphragmatic paresis (DP) within the immediate postoperative period.

## Objectives

Primary: Evaluate the association between DP and respiratory infections occurring in the postoperative period of LT. Secondary: Assess the impact on outcomes of DP in LT recipients.

## Methods

We prospectively followed all the adult patients who were consecutively admitted to the intensive care unit (ICU) after LT between September 2011 and September 2014. A thoracic echography was performed to all the patients with clinical criteria for diaphragmatic weakness. This was clinically suspected when weaning from the ventilator failed. The diaphragm function was evaluated in the M mode, diagnosing as diaphragmatic paresis all patients with a diaphragm excursion inferior to 9 mm (women) or 10 mm (men), or with a thickening fraction [(thickness at end-inspiration - thickness at end-expiration)/thickness at end-expiration] less to 20%, also qualitative discrimination were made between reduced and paradoxical inspiratory movement (1). We studied the association of confirmed diaphragmatic paresis with pneumonia and tracheobronchitis (2) as primary endpoints. Secondary endpoints were incidence of tracheostomy, days on the ventilator and in ICU and hospital mortality. Continuous data are reported as median and interquartile range and categorical data as numbers and percentages. Comparison was done using odds ratio (OR) and 95% confidence interval (CI), with absence of DP as reference.

## Results

Our cohort comprised 187 LT recipients. Median age was 57 (51.5-62.5) and 55.6% got bilateral LT. Thirteen patients were excluded due to impossibility to evaluate the diaphragmatic function. In 94 (54.02%) patients DP was clinically suspected and lung ultrasound confirmed it in 59 (33.90%). When compared with the rest of the population, these patients suffered more episodes of tracheobronchitis (OR 2.5; CI 5.8-1.1) but not pneumonia (OR 1.7; CI 4.8-0.5). They were more frequently tracheostomized (OR 5.6; CI 12.8-2.6) and stayed more days on the ventilator, in ICU and in hospital. However, we found no differences on ICU mortality (OR 1.3; CI 5.8-0.2). Details are shown in the flowchart.

## Conclusion

Using lung ultrasound, DP was identified in one-third of the LT recipients in the ICU postoperative period. It was associated with increased use of healthcare resources but not with early mortality. Quality improvement efforts should be addressed to reduce the incidence of DP and improve ICU management.

**Figure 1:**
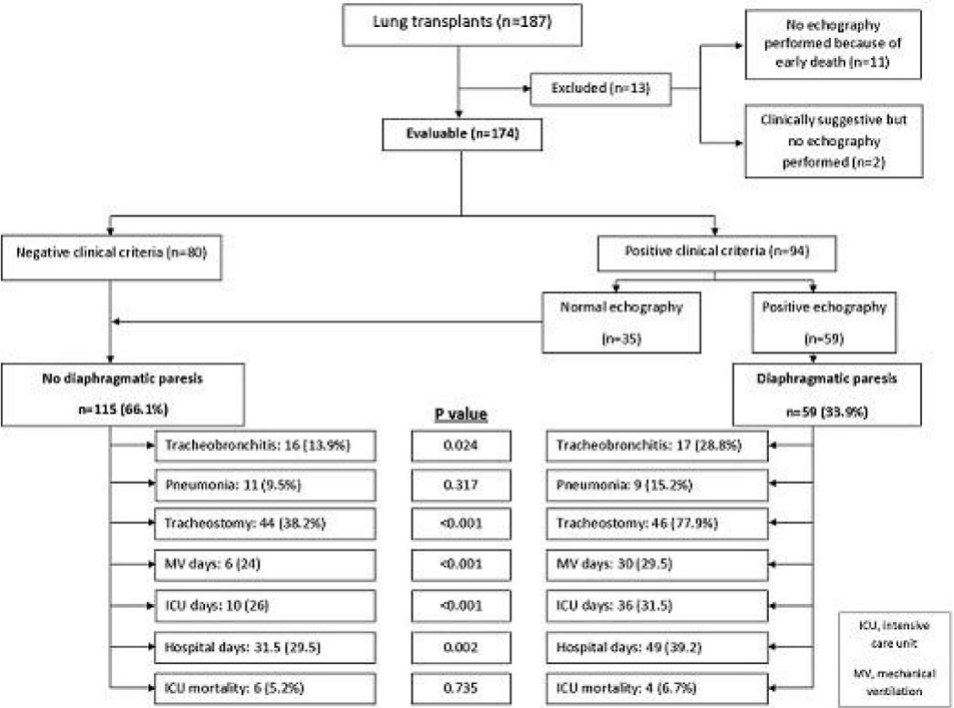
**Diagram of patient flow and comparison of outcomes**.

